# The efficacy of tranexamic acid treatment with different time and doses for traumatic brain injury: a systematic review and meta-analysis

**DOI:** 10.1186/s12959-022-00440-9

**Published:** 2022-12-19

**Authors:** Honghao Huang, Mei Xin, Xiqiang Wu, Jian Liu, Wenxin Zhang, Ke Yang, Jinbao Zhang

**Affiliations:** 1 Department of Cardiovascular Surgery, General Hospital of Western Theater Command (Chengdu Military General Hospital), Chengdu, 610036 China; 2 College of Medicine, Southwest Jiaotong University, Chengdu, 610036 China

**Keywords:** Brain injury, Clinical trial, Randomized, Tranexamic acid, Traumatic

## Abstract

**Objective:**

Tranexamic acid (TXA) plays a significant role in the treatment of traumatic diseases. However, its effectiveness in patients with traumatic brain injury (TBI) seems to be contradictory, according to the recent publication of several meta-analyses. We aimed to determine the efficacy of TXA treatment at different times and doses for TBI treatment.

**Methods:**

PubMed, MEDLINE, EMBASE, Cochrane Library, and Google Scholar were searched for randomized controlled trials that compared TXA and a placebo in adults and adolescents (≥ 15 years of age) with TBI up to January 31, 2022. Two authors independently abstracted the data and assessed the quality of evidence.

**Results:**

Of the identified 673 studies, 13 involving 18,675 patients met our inclusion criteria. TXA had no effect on mortality (risk ratio (RR) 0.99; 95% confidence interval (CI) 0.92–1.06), adverse events (RR 0.93, 95% Cl 0.76–1.14), severe TBI (Glasgow Coma Scale score from 3 to 8) (RR 0.99, 95% Cl 0.94–1.05), unfavorable Glasgow Outcome Scale (GOS < 4) (RR 0.96, 95% Cl 0.82–1.11), neurosurgical intervention (RR 1.11, 95% Cl 0.89–1.38), or rebleeding (RR 0.97, 95% Cl 0.82–1.16). TXA might reduce the mean hemorrhage volume on subsequent imaging (standardized mean difference, -0.35; 95% CI [-0.62, -0.08]).

**Conclusion:**

TXA at different times and doses was associated with reduced mean bleeding but not with mortality, adverse events, neurosurgical intervention, and rebleeding. More research data is needed on different detection indexes and levels of TXA in patients with TBI, as compared to those not receiving TXA; although the prognostic outcome for all harm outcomes was not affected, the potential for harm was not ruled out.

**Trial registration:**

The review protocol was registered in the PROSPERO International Prospective Register of Systematic Reviews (CRD42022300484).

**Supplementary Information:**

The online version contains supplementary material available at 10.1186/s12959-022-00440-9.

## Background

Traumatic brain injury (TBI) is an organic injury of brain tissue caused by external violence [1]. More than 50 million people worldwide suffer from TBI every year, and approximately half of the world's population may suffer from TBI once or more in their lifetime [2]. The annual incidence rate of TBI in Europe ranges from 47.3/10 million to 849/10 million, with a mortality of 3.3/10 million to 28.1/10 million [3]. The high mortality rate associated with TBI may be closely related to its complex pathophysiology.

TBI includes initial head trauma via an external force that results in mechanical damage to brain tissue, and subsequent biochemical cascades such as apoptosis, mitochondrial dysfunction, cortical spreading depression, and microvascular thrombosis [4]. As a result, nerve damage in differing proportions inevitably occurs, with various resultant clinical courses occurring during this process, including intracranial hematoma, brain tissue contusion, and cerebral ischemia [5]. In particular, intracranial hematoma in half of the patients increases after hospital admission, which increases the difficulty of surgical removal and leads to high mortality and disability [6].

Currently, the treatment of TBI mainly involves hyperosmolar therapy, seizure prophylaxis, medically induced comatose state, invasive intracranial monitoring, and radical decompressive surgical interventions as a last resort [7]. These methods, except for surgical interventions, may achieve symptom relief in a short time but do not address the prognosis of TBI. Therefore, research on the treatment of this disease is required, especially regarding hemostatic drugs designed to protect against long-term damage from TBI.

Recently, tranexamic acid (TXA), a synthetic lysine derivative, was shown to play important roles in the treatment of traumatic diseases [8]. TXA exerts its hemostatic function by competitively occupying the lysine binding site of plasminogen, thereby blocking its interaction with fibrin and subsequent clot breakdown [9]. Extensive trials conducted in patients with severe trauma with massive bleeding using TXA have demonstrated that survival increased when TXA was administered early after an accident compared with a placebo [10]. However, the role of TXA in patients with TBI or intracranial bleeding remains controversial [11]. After the recent systematic review and meta-analysis, which included 5 multi-site RCTs and 8 single-site RCTs (The specific information of the RCT is shown in Table 1) [12], we performed this meta-analysis of all related articles to examine the effectas of TXA in TBI patients..

**Table 1:** Baseline characteristics of included studies

Study author and year	Study design	patients TXA/placebo	TXA dose	Male (%)	Mean or median age in years TXA/placebo	Enrollment time after trauma	Inclusion criteria	Exclusion criteria
Roberts et al. 2019	Multisite RCT	6406/6331	1 g TXA bolus followed by 1 g TXA maintenance	TXA: 3742 (80%) placebo: 3660 (80%)	TXA: 41.7 (19) placebo: 41.9 (19)	2 h	(1) Adults with TBI within 3 h of injury (2) GCS ≤ 12 or any intracranial bleeding on CT scan	Major extracranial bleed
Rowell et al. 2020 [13]	Multisite RCT	657/309	Bolus-Maintenance arm: 1 g TXA bolus followed by 1 g TXA. Bolus only arm: 2 g TXA bolus followed by a placebo infusion	Bolus-Maintenance: 227 (73%) Bolus-Only: 255 (74%) placebo: 233 (75%)	Bolus-Maintenance arm: 39(26–57) Bolus Only arm: 40(26–56) Placebo arm: 36(25–55)	2 h	(1) GCS ≤ 12 (2) Prehospital SBP ≥ 90 (3) Age ≥ 15 years (or weight ≥ 50 kg if age is unknown)	(1) GCS = 3 with unreactive pupil (2) CPR by EMS prior to randomization (3) Burns (4) Pregnancy
Mahmood et al. 2021 [14]	Multisite RCT	884/883	1 g TXA bolus followed by 1 g TXA maintenance	TXA: 701 (79%) placebo: 712 (81%)	TXA: 45 (29–64) placebo: 45 (29–63)	3 h	(1) Adults with head injury who were within 3 h of injury; (2) Glasgow Coma Score (GCS) of ≤ 12; (3) any intracranial bleeding on CT, and no significant extracranial bleeding	significant extracranial bleeding
van Wessem et al. 2021 [15]	Single site RCT	120/114	1 g TXA bolus followed by 1 g TXA maintenance	TXA: 80 (67%) placebo: 77 (68%)	TXA: 42 (23–59) placebo: 53 (33–65)	1 h	TBI (AIS head ≥ 3) who were admitted to the adult ICU	AIS head scores based on isolated C-spine injuries
Mojallal et al. 2020 [16]	Single site RCT	56/44	1 g TXA bolus followed by 1 g TXA maintenance	TXA: 40 (71.4%) placebo: 40 (90.9%)	N/A	8 h	(1) age > 18. (2) detection of cerebral hemorrhage in brain CT scan (3) passage of less than 8 h after trauma incidence (4) negative history of taking anticoagulants (5) negative history of blood coagulation system impairments	patients who underwent craniotomy less than 24 h
Mousavinejad et al. 2020 [17]	Single site RCT	20/20	1 g TXA bolus followed by 1 g TXA maintenance	TXA: 6 (30%) placebo: 8 (40%)	55 ± 19/55 ± 18	8 h	(1) ≥ 18 years within 8 h of injury (2) TBI on brain CT with no significant epidural hemorrhage (3) The need for surgery	(1) Pregnancy (2) Coagulopathy (3) Massive transfusion and/or fresh frozen plasma (FFP)
Yutthakasemsunt et al. 2013 [18]	Single site RCT	120/118	1 g TXA bolus followed by 1 g TXA maintenance	TXA: 103 (86%) placebo: 107 (91%)	35 (16)/ 34 (15)	8 h	(1) Age ≥ 16 years (2) Moderate to severe TBI (GCS) 4 to 12 (3) Had a CT brain within 8 h (4) No immediate indication for surgery	(1) Immediate need for surgery (2) Coagulopathy (3) Known to be receiving a medication that affects hemostasis (4) Pregnancy
Fakharian et al. 2017	Single site RCT	78/78	1 g TXA bolus followed by 1 g TXA maintenance	TXA: 67 (90.5) placebo: 6 688)	42 ± 18/39 ± 18	8 h	(1) Age ≥ 15 years (2) Non-penetrating injury and any kind of Traumatic ICH (3) Arrived at the hospital within 8 h 4) No need for brain surgery during the first 8 h	(1) Major organ damage (2) Pregnancy (3) Receiving any medication that disturbs homeostasis (4) Coagulopathy
Jokar et al. 2017 [19]	Single site RCT	40/40	1 g TXA bolus followed by 1 g TXA maintenance	TXA: 32 (40.0%) placebo: 28 (35.0%)	35 ± 15/ 36 ± 145	2 h	(1) TBI patients aged 15 years and more (2) Within 2 h of injury onset (3) Acute ICH (volume of less than 30 ml) based on CT scan findings	(1) GCS < 8 (2) Need for surgery (3) Cerebral edema with midline shift (4) Coagulation disorders (5) Pregnancy (6) History or current VTE
Ebrahimi et al. 2019 [20]	Single site RCT	40/40	1 g TXA bolus followed by 1 g TXA maintenance	SDH-TXA: 17 (85%) SDH-placebo: 17 (85%) EDH-TXA: 16 (80%) EDH-placebo: 18 (90%)	SDH: 40 ± 18/40 ± 18 EDH: 24 ± 7/25 ± 7	8 h	(1) Adults within 8 h of injury (2) Isolated SDH or EDH requiring surgery	(1). Major extracranial bleeding (2) Massive transfusion (3) Coagulopathy (4) Pregnancy
Perel et al. 2012 [21]	Multisite RCT	133/137	1 g TXA bolus followed by 1 g TXA maintenance	TXA: 111 (84.0) placebo: 117 (85.0)	36.2 (14.0)/37.0 (13.7)	8 h	1) Fulfills the inclusion criteria for the CRASH-2 trial (2) GCS ≤ 14 (3) Baseline clinical CT scan consistent with TBI	(1) Pregnancy and (2) Patients for whom a second brain CT scan was not possible
Bossers et al. 2021 [22]	Multisite RCT	693/1134	1 g TXA bolus followed by 1 g TXA maintenance: 615 > 2 g TXA bolus: 4	TXA: 486 (70%) placebo: 797 (70%)	47 (25–66)/45 (22–65)	N/A	1) severe TBI, GCS ≤ 8 2) suspected rather than confirmed TBI because prehospital treatment, including administration of tranexamic acid	1) BRAIN-PROTECT database who were not transported to a participating trauma center (no follow-up data were available) 2) undergoing prehospital traumatic cardiopulmonary resuscitation (inherently very high mortality, regardless of treatment)
Chakroun et al. 2018	Single site RCT	96/84	1 g TXA bolus followed by 1 g TXA maintenance	TXA: 88 (91.7%) placebo: 797 (70%)	44 ± 20/39 ± 18	8 h	(1) age > 18. (2) intracranial bleeding in the first or the second brain CT-scan (3) a delay of management in the study center under 24 h after trauma	1) significant extra cranial bleeding 2) TXA can improve outcome

*RCT* Randomized-controlled trial, *TXA* Tranexamic acid, *GCS* Glasgow coma scale, *GOS* Glasgow outcome scale, *TBI* Traumatic brain injury, *SDH* Subdural hemorrhage, *EDH* Epidural hemorrhage, *ICH* Intracranial hemorrhage, *N/A* Not applicable

## Methods

This systematic review and meta-analysis was conducted based on the Cochrane Handbook for Systematic Reviews of Interventions [23] and the “Preferred Reporting Items for Systematic Reviews and Meta-Analysis” recommendations [24]. Two investigators independently searched for articles, extracted the data, and assessed the quality of the included studies.

### Search strategy

PubMed, MEDLINE, EMBASE, Cochrane Library, and Google Scholar were searched for RCTs that compared TXA and a placebo in adults and adolescents (≥ 15 years of age) with TBI, up to January 31, 2022. With the assistance of an expert medical librarian, we developed a search strategy, including three search terms: "tranexamic acid,” "traumatic brain injury" and "randomized controlled trial" (appendix 1–1). We also searched the proceedings of emergency medicine, hematology, trauma, neurology, and neurosurgery conferences to identify relevant abstracts.

### Study selection

We included all English literature on RCTs on the treatment of TBI with TXA and performed a meta-analysis. Our inclusion criteria were as follows: (1) studies conducted in patients with TBI receiving any dose of TXA. 2) Patients with any type of intracranial hemorrhage secondary to TBI. The exclusion criteria were as follows: 1) preclinical study; 2) The research type was repeated reports or published studies such as case reports, reviews, or literature without control, focusing on surgical methods, surgical techniques, and imported instruments; 3) study with nonclinical patients such as animals, corpses, or specimens; 4) non-English-language publications; and 5) repetitive studies.

### Data extraction and quality assessment

Two authors independently abstracted the data and assessed the quality of evidence. We extracted the following information from the included studies [13–22, 25–27]: study author and year of study, study design, demographic data, age and sex of the participants, details of the intervention, and risk of bias. We extracted the results of the included studies as follows: mortality, severe TBI (Glasgow Coma Scale score from 3 to 8), unfavorable Glasgow Outcome Scale (GOS < 4), neurosurgical intervention, mean hemorrhage volume, the number of patients with rebleeding, adverse events including vascular occlusive events, pulmonary embolism, deep vein thrombosis, neurological complications (including stroke and seizure), gastrointestinal bleeding, myocardial infarction, infectious complications, and renal failure. The Cochrane risk of bias assessment tool (Cochrane Collaboration) was used to assess the quality of the included trials and rate the level of evidence.

### Statistical analysis

We used RevMan 5.3 software provided by the Cochrane Collaboration Network for meta-analysis, and Stata 16.0 software for Harbord’s test and Egger’s test. Dichotomous data were measured using risk ratios (RR) and 95% confidence interval (CI). In this analysis, because the average blood loss units in some studies was inconsistent with that in other studies, continuous variables were measured using standardized mean difference (SMD) and 95% Cl. Heterogeneity among the included studies was examined using the chi-square and I^2^ tests. When there was statistical homogeneity among the results (P ≥ 0.1, I^2^ < 50%), the fixed-effects model was used to continue the meta-analysis. If there was statistical heterogeneity among the research results (*P* < 0.1, I^2^ > 50%), the random-effects model was used for the meta-analysis. We did not construct funnel plots to assess publication bias, as these were inaccurate when fewer than 10 trials were included in the analysis. Publication bias of the included studies was analyzed using Harbord’s test and Egger’s test.

## Result

### Literature search

**Fig. 1:**
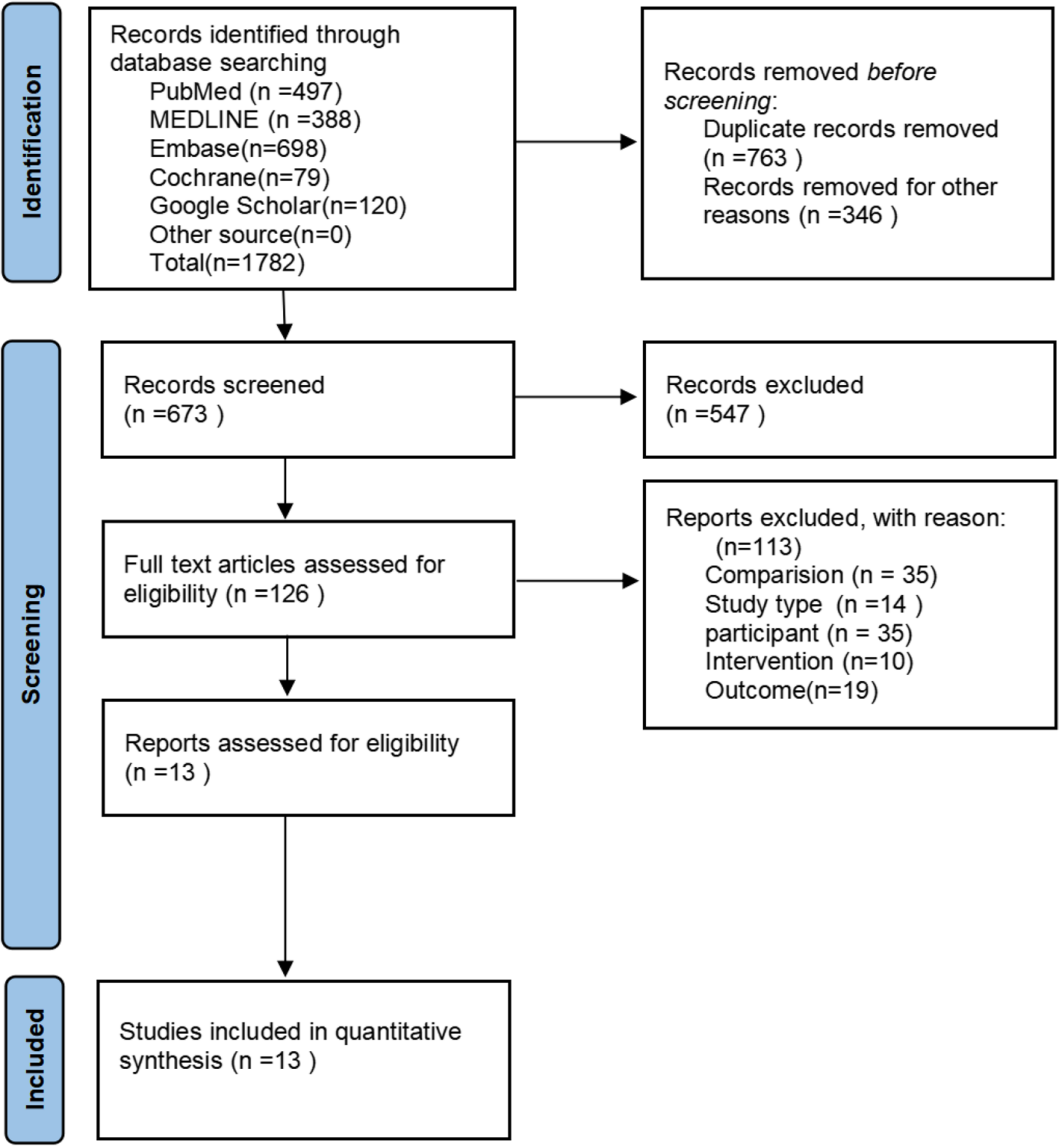
Flow diagram of study selection process for systematic review

Based on the results of the search strategy results, 1782 relevant articles were screened. After excluding duplicated articles and those that met the exclusion criteria described in Sect. 2.2, 673 articles remained. After reading the title and abstract according to the inclusion criteria, 547 articles were excluded, yielding 126 studies. The full text of these articles was assessed, leading to the exclusion of another 113 studies, resulting in 13 studies with 18,675 patients, which were included in the analysis [13–22, 25–27]. The baseline characteristics of the included studies are summarized in Table 1. A flowchart of the literature search strategy is presented in Fig. 1.

### Description of included studies

**Fig. 2:**
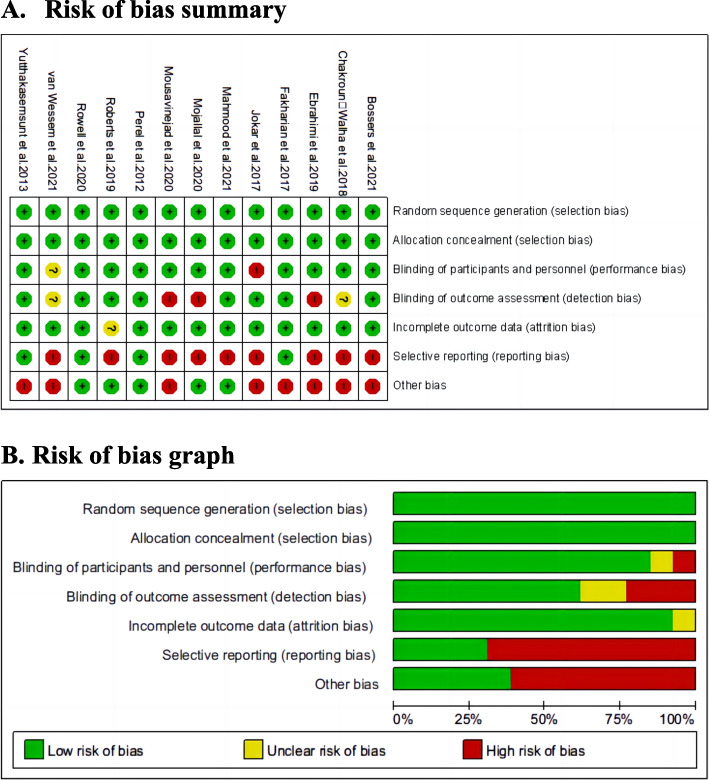
Risk of bias assessment. **A**. Authors' judgments about each risk of bias item for each included study. **B**. Authors' judgments about each risk of bias item presented as percentages across all included studies

Thirteen articles were included, including eight single-site RCTs [15–20, 26, 27] and five multi-site RCTs [13, 14, 21, 22, 25]. The minimum age of the participants was greater than 15 years, which included both adults and adolescents. The timing of TXA administration varied among studies, with five trials in which the post-traumatic registration time was less than 3 h [13, 15, 17, 19, 25], seven trials in which the post-traumatic registration time was more than 3 h [14, 16, 18, 19, 21, 26, 27], and one article that did not clearly explain the registration time. In the included trials, the TXA dose was mostly similar, and the most common regimen was a loading dose of 1 g, followed by a maintenance dose of 1 g over 8 h [13–22, 25–27]. However, one trial used a loading dose of 2 g followed by a maintenance dose of 2 g over 8 h [13]. The risk of bias was lower in eight articles and higher in five articles and is shown in Fig. 2. The regression-based Harbord’s and Egger’s tests were not statistically significant for all outcomes (Table 2). Since the mean hemorrhage volume is continuous data, Harbord’s test could not be utilized. Gastrointestinal bleeding (*n* = 2), myocardial infarction (*n* = 2), infection complications (*n* = 2), and renal failure (*n* = 1) were included in the less relevant articles; therefore, they were not tested.

**Table 2:** The risk of bias of studies

Outcomes	Heterogeneity (I^2^), *P*-value	Harbord’s test	Egger’s test	number of studies
Mortality	48%, 0.04	0.4865	0.4832	10
severe TBI (GCS 3 to 8)	0%, 0.79	0.3983	0.3346	4
GOS < 4	71%, 0.004	0.5533	0.5925	6
Neurosurgical Intervention	0%, 0.73	0.3403	0.3356	5
Rebleeding	0%, 0.73	0.5421	0.5066	4
adverse event^a^	50%, 0.08	0.1543	0.2468	6
Vascular occlusive events	0%, 0.37	0.8878	0.9214	3
Pulmonary embolism	64%, 0.06	0.2980	0.7334	4
Deep vein thrombosis	0%, 0.60	0.6228	0.8206	4
Neurological complications	27%, 0.25	0.0328	0.0682	4

*TBI* Traumatic brain injury, *GCS* Glasgow coma scale, *GOS* Glasgow outcome scale

^a^indicates Vascular occlusive events + Pulmonary embolism + Deep vein thrombosis + Neurological complications

### Prognostic outcome

**Fig. 3:**
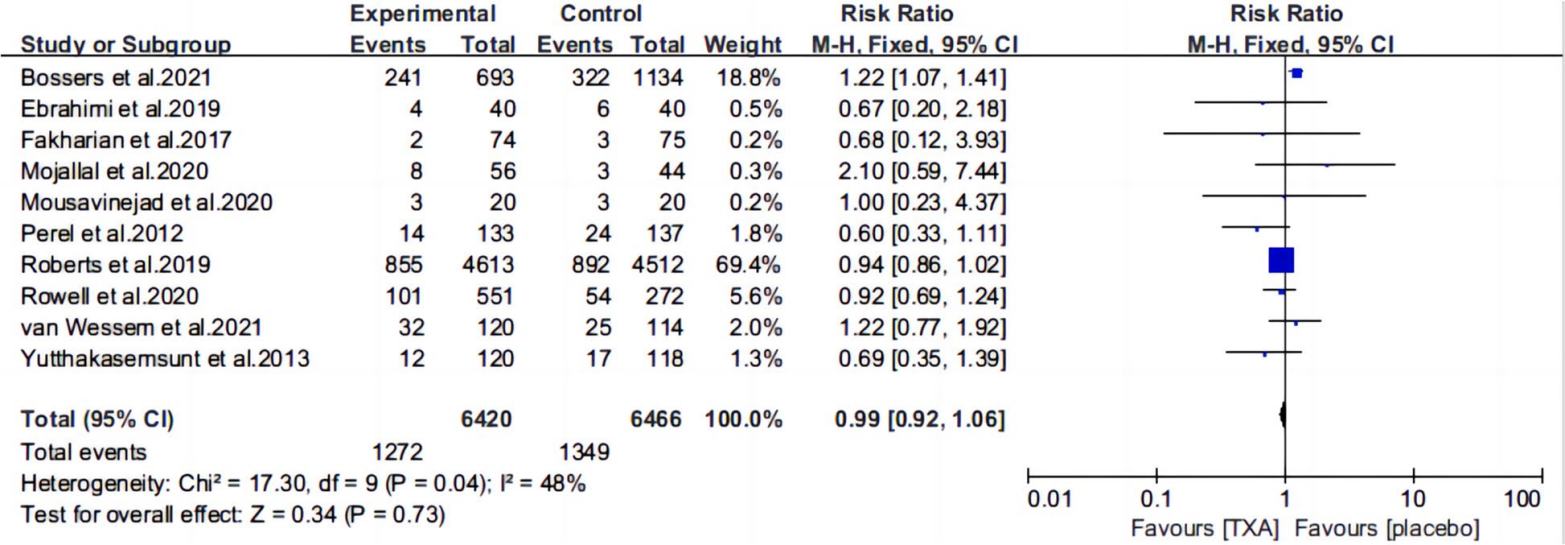
Forest plot comparing TXA and placebo for the outcome of all-cause mortality

**Fig. 4:**
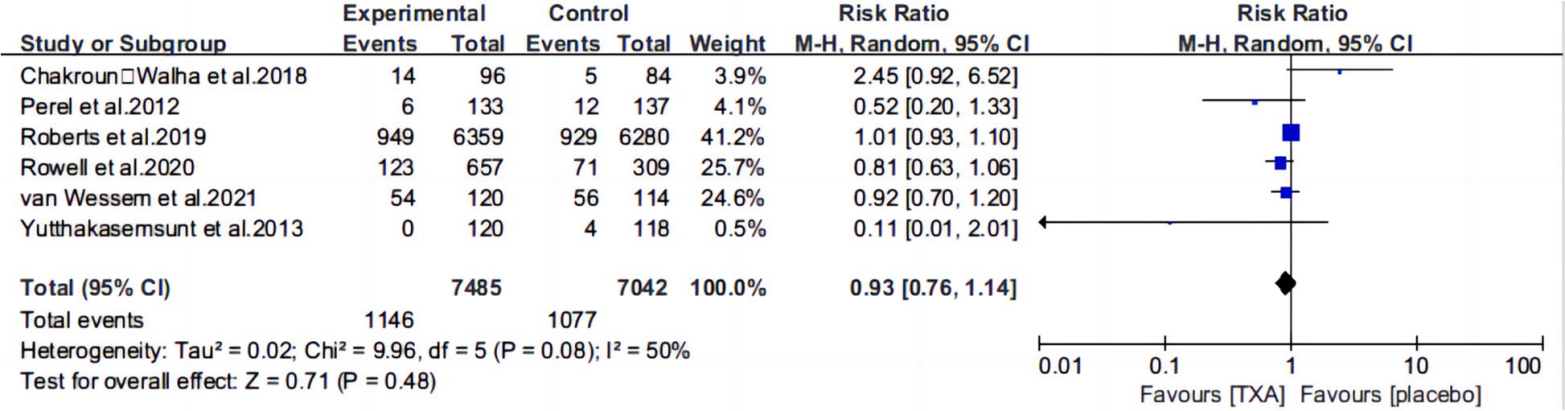
Forest plot comparing TXA and placebo for all adverse events

**Fig. 5:**
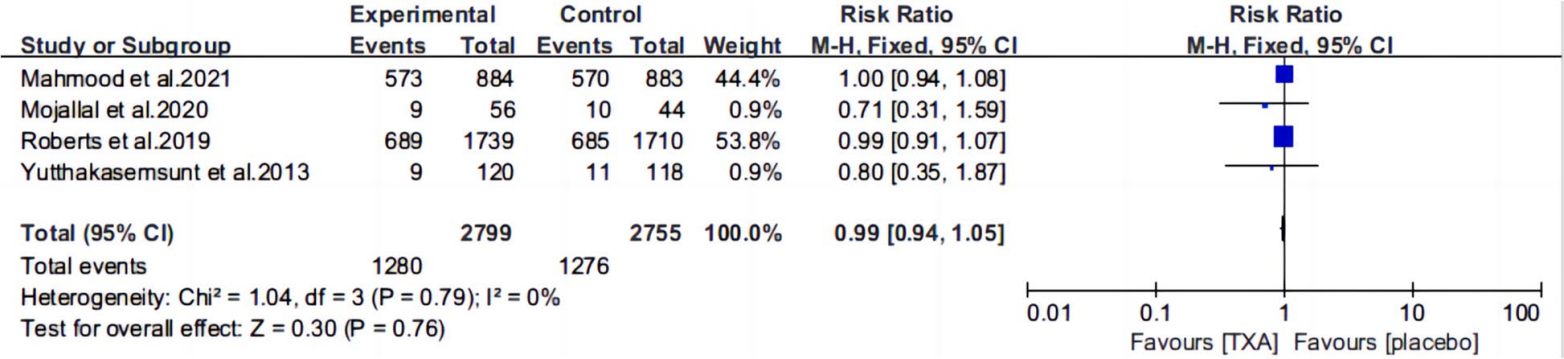
Forest plot comparing TXA and placebo for severe TBI (Glasgow Coma Scale) score from 3 to 8)

**Fig. 6:**
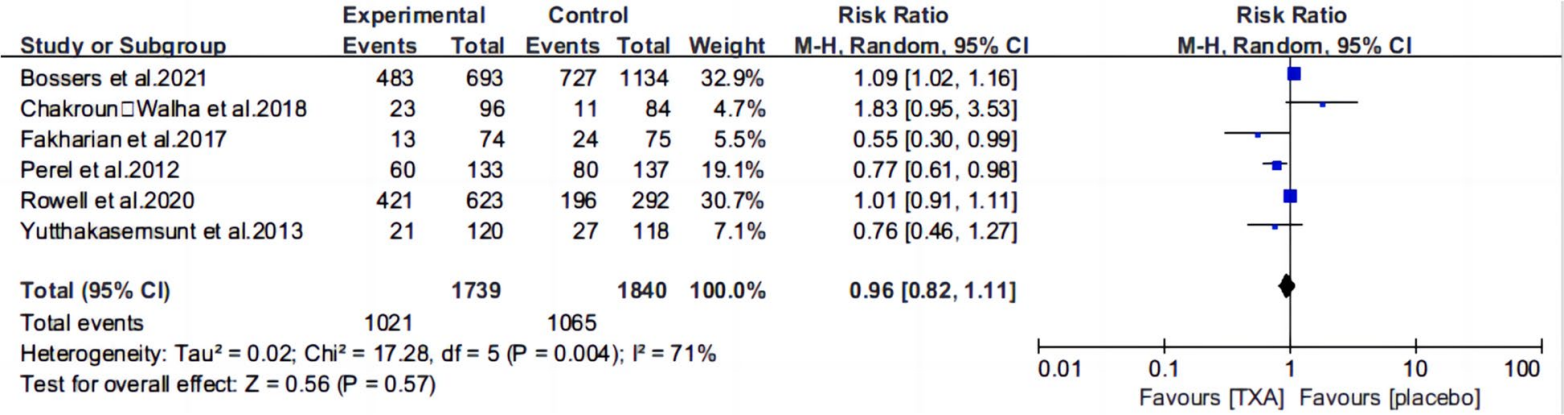
Forest plot comparing TXA and placebo for the unfavorable Glasgow Outcome Scale (GOS < 4)

**Fig. 7:**
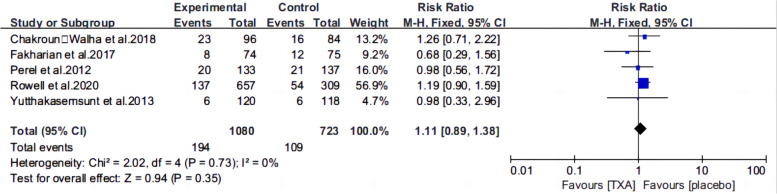
Forest plot comparing TXA and placebo for the need of neurosurgical intervention

**Fig. 8:**
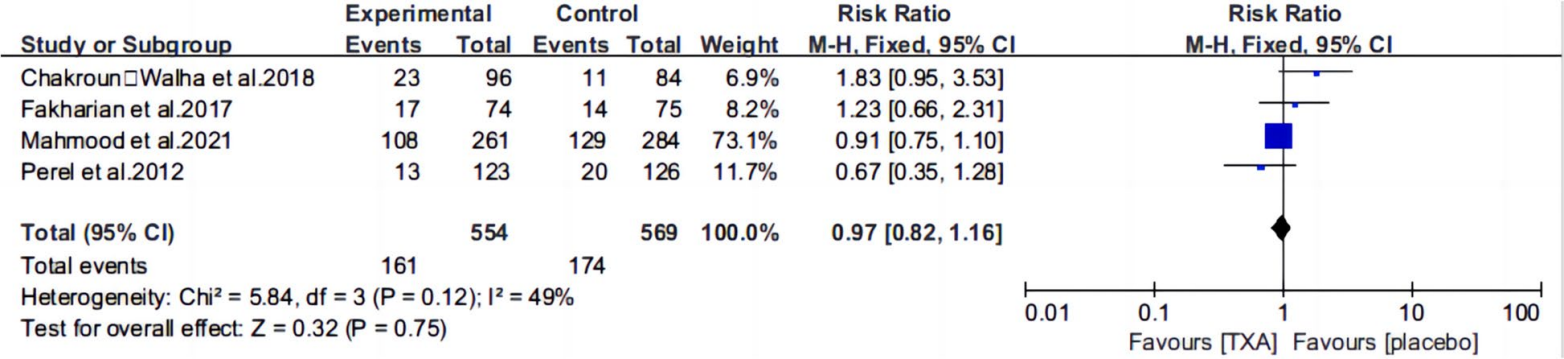
Forest plot comparing TXA and placebo for the number people of Rebleeding

**Fig. 9:**
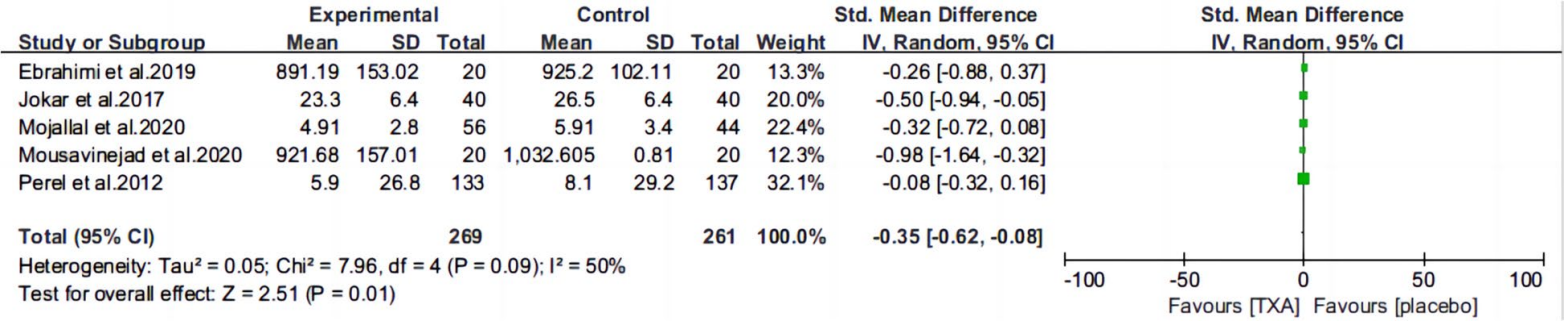
Forest plot comparing TXA and placebo for mean hemorrhage volume

Ten articles [13, 15–22, 25, 26] reported the mortality of patients with TBI treated with TXA or a placebo. Meta-analysis showed that TXA was not associated with reduced mortality (RR 0.99; 95% CI 0.92–1.06, Fig. 3), adverse events (RR 0.93, 95% Cl 0.76–1.14, Fig. 4), severe TBI (Glasgow Coma Scale score from 3 to 8) (RR 0.99, 95% Cl 0.94–1.05, Fig. 5), unfavorable Glasgow Outcome Scale (GOS < 4) (RR: 0.96, 95% Cl: 0.82–1.11, Fig. 6), neurosurgical intervention (RR 1.11, 95% Cl 0.89–1.38, Fig. 7), or the number instances of rebleeding (RR 0.97, 95% Cl 0.82–1.16, Fig. 8). Our meta-analysis showed that TXA may reduce mean hemorrhage volume in TBI patients on subsequent imaging (SMD -0.35; 95% CI [-0.62, -0.08], Fig. 9). Two articles [17, 19] showed that TXA was a protective factor for mean hemorrhage volume, and three [16, 20, 21] showed that TXA was not associated with mean hemorrhage volume.

### Safety

**Fig. 10:**
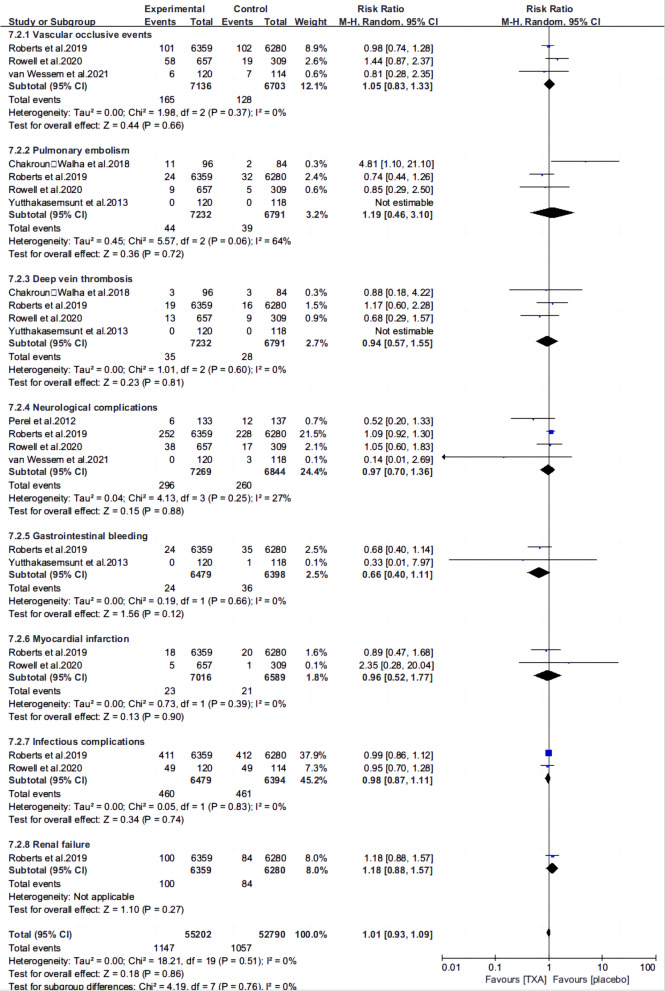
Forest plot comparing TXA and placebo for adverse events of various causes

We found similar rates of adverse events [13, 15, 18, 21, 25, 27] between those receiving and those not receiving TXA (RR 0.93, 95% Cl 0.76–1.14). We conducted an in-depth analysis to understand the impact of TXA on different adverse events (Fig. 10). Pooled results demonstrated no increased risk of vascular occlusive events (RR 1.05, 95% CI 0.83–1.33), pulmonary emboli (RR 1.19, 0.46–3.10), deep vein thrombosis (RR 0.94, 95% CI 0.57–1.55), neurological complications (RR 0.97, 95% CI 0.70–1.30), gastrointestinal bleeding (RR 0.66, 95% Cl 0.40–1.11), myocardial infarction (RR 0.96 95% Cl 0.52–1.77), infectious complications (RR 0.98, 95% Cl 0.87–1.11), or renal failure (RR 1.18, 95% Cl 0.88–1.57) in patients receiving TXA, as compared to those not receiving TXA, although confidence intervals for all harm outcomes were wide, and did not rule out the potential for harm.

### Sensitivity and subgroup analysis

We performed a subgroup analysis of the study design (multi- or single-site RCT), enrollment time after trauma (< 3 h or > 3 h), and TXA dose (2 g TXA bolus followed by placebo infusion or 1 g TXA bolus followed by 1 g TXA maintenance). Table 3 presents the results of the analysis. None of the subgroup analyses showed differences in estimates or conclusions for any of the outcomes of interest appendix 1–2).

**Table 3:** Subgroup Analysis of TXA and placebo

	**Mortality**		**Adverse Events**		**Neurosurgical Intervention**		**Rebleeding**		**Mean hemorrhage volume**	
	RR, 95%	*P*-Value	RR, 95%	*P*-Value	RR, 95%	*P*-Value	RR, 95%	*P*-Value	RR, 95%	*P*-Value
Study design										
Multisite RCT	0.99 [0.79–1.24]	0.90	0.91 [0.73–1.13]	0.38	1.01 [0.61–1.55]	0.96	0.81 [0.59–1.10]	0.17	-0.08 [-0.32, 0.16]	0.52
Single site RCT	1.03 [0.73–1.43]	0.88	1.07 [0.39–2.88]	0.90	1.15 [0.89–1.48]	0.29	1.65 [0.94–2.89]	0.88	-0.46 [-0.72, -0.20]	0.0005
Enrollment time after trauma										
< 3 h	0.94 [0.87–1.02]	0.15	0.91 [0.77–1.08]	0.28	1.18 [0.89–1.55]	0.24	0.91 [0.75–1.10]	0.34	-0.50 [-0.94, -0.05]	0.03
> 3 h	0.76 [0.51–1.11]	0.15	2.45 [0.92–6.52]	0.07	1.00 [0.70–1.44]	0.99	1.14 [0.65–2.02]	0.64	-0.33 [-0.65, 0.00]	0.05
TXA dose										
2 g TXA bolus followed by a placebo infusion	0.80 [0.56–1.15]	0.22	1.22 [0.85–1.74]	0.28	N/A	N/A	1.24 [0.91–1.70]	0.17	N/A	N/A
1 g TXA bolus followed by 1 g TXA maintenance	1.01 [0.82–1.24]	0.95	1.00 [0.92–1.08]	0.97	N/A	N/A	1.00 [0.71–1.41]	1	N/A	N/A

*N/A* Not applicable

We divided the study design into single-site RCT [15–20, 26, 27] and multi-site RCT [13, 14, 21, 22, 25]. TXA had no effect on mortality (RR 0.99, 95% Cl 0.79–1.24, P 0.90), adverse events (RR 0.91, 95% Cl 0.73–1.13, P 0.38), neurosurgical intervention (RR 1.01, 95% Cl 0.61–1.55, P 0.96), rebleeding (RR 0.81, 95% Cl 0.59–1.10, P 0.17), or mean hemorrhage volume (RR -0.08, 95% Cl [-0.32, 0.16], P 0.52) in multi-site RCTs. TXA had no effect on mortality (RR 1.03, 95% Cl 0.73–1.43, P 0.88), adverse events (RR 1.07, 95% Cl 0.39–2.88, P 0.90), neurosurgical intervention (RR 1.15, 95% Cl 0.89–1.48, P 0.29), rebleeding (RR 1.65, 95% Cl 0.94–2.89, P 0.88), or mean hemorrhage volume (RR -0.46, 95% Cl [-0.72, 0.20], P 0.0005) in single-site RCTs.

We observed the effect of TXA on prognosis according to the enrollment time after trauma. When the enrollment time after trauma was less than 3 h [13, 15, 17, 19, 25], TXA had no effect on mortality (RR 0.94, 95% Cl 0.87–1.02, P 0.15), adverse events (RR 0.91, 95% Cl 0.77–1.08, P 0.28), neurosurgical intervention (RR 1.18, 95% Cl 0.89–1.55, P 0.24), or rebleeding (RR 0.91, 95% Cl 0.75–1.10, *P* = 0.34), but reduced mean hemorrhage volume (RR -0.50, 95% Cl [-0.94, -0.05], P 0.03). When enrollment time after trauma was greater than 3 h [14, 16, 18, 20, 21, 26, 27], TXA had no effect on mortality (RR 0.76, 95% Cl 0.51–1.11, P 0.15), adverse events (RR 2.45, 95% Cl 0.92–6.52, P 0.07), neurosurgical intervention (RR 1.00, 95% Cl 0.70–1.44, P 0.99), rebleeding (RR 1.14, 95% Cl 0.65–2.20, P 0.34), or mean hemorrhage volume (RR -0.33, 95% Cl [-0.65, 0.00], P 0.05).

Different doses of TXA had no effect on adverse events. TXA [13] had no effect on mortality (RR 0.80, 95% Cl 0.56–1.15, P 0.22), adverse events (RR 1.22, 95% Cl 0.85–1.74, P 0.28), or rebleeding (RR 1.24, 95% Cl 0.91–1.70, P 0.17) in 2 g TXA bolus followed by a placebo infusion. TXA [13–22, 25–27] had no effect on mortality (RR 1.01, 95% Cl 0.82–1.24, P 0.95), adverse events (RR 1.00, 95% Cl 0.92–1.08, P 0.97), or rebleeding (RR 1.00, 95% Cl 0.71–1.41, P 1) in 1 g TXA bolus followed by 1 g TXA maintenance.

## Discussion

TBI is a serious threat to human health and has attracted research interest owing to its high mortality rate [28]. However, owing to its complex pathophysiology, the treatment of TBI has posed a problem for clinicians and researchers [29]. Recently, TXA, a drug used to reduce bleeding for various indications, has been shown to play an important role in the treatment of TBI. However, the efficacy of TXA at various times and doses remains unclear. We will discuss the hemostatic effect, mortality, and adverse events in patients with TBI treated with TXA compared with a placebo at different times and doses.

### Hemostatic effect of TXA with respect to time and dose

The coagulopathy of TBI generally does not provoke hyperfibrinolysis and can even result in an acute impairment of fibrinolysis, referred to as fibrinolytic shutdown [30]. A previous study demonstrated that delayed TXA for TBI has been shown to enhance fibrinolysis via the urokinase plasminogen activator [31]. However, another study showed that TBI may lead to hyperfibrinolysis under specific conditions [30]. Therefore, coagulopathy associated with extracranial injuries is primarily caused by substantial blood loss (hemorrhagic shock), consumption, hypothermia, and hypoperfusion-induced metabolic acidosis, which can be further propagated by iatrogenic factors, such as fluid resuscitation (hemodilution) [32, 33]. Hyperfibrinolysis appears to be closely associated with lethal hemorrhagic shock and is relatively independent of injury severity, which was corroborated in an animal model where isolated hemorrhagic shock induced tissue plasminogen activator-mediated hyperfibrinolysis, whereas isolated tissue injury reduced fibrinolytic activity [34, 35]. Secondary infection after hemorrhage is also one of the factors that promote death in patients [36]. TXA with antifibrinolytic and anti-inflammatory properties is effective in avoiding the progression of hemorrhage volume and controlling its associated inflammation in traumatized patients.

This meta-analysis demonstrated that TXA had no effect on rebleeding but reduced the mean hemorrhage volume on subsequent imaging. This result is different from those of previous studies, which indicated that TXA can inhibit rebleeding after TBI, and the possible benefits of TXA appear in specific populations [37, 38]. For example, patients with moderate and severe hypertension may achieve a better inhibitory effect on rebleeding using TXA. Due to the lack of data related to blood pressure, we could not analyze it in depth.

Our subgroup analysis showed that the timing and dose of TXA were not risk factors for re-bleeding. The result of mean hemorrhage volume was consistent with the CRASH-2 trial, that is, administration of TXA within 8 h was not associated with the mean bleeding volume (RR -0.33, 95% Cl [-0.65, 0.00], P 0.05) [39]. Our subgroup analysis showed that the timing of TXA administration within 3 h after injury could reduce the mean hemorrhage volume but had no effect beyond 3 h after injury. Therefore, the timing of TXA administration is one of the factors affecting the hemostatic effects.

### Mortality after the treatment of TXA with respect to time and dose

This meta-analysis demonstrated that TXA has no obvious effect on mortality. This disagrees with other meta-analyses performed at an earlier stage. Some studies have demonstrated a reduction in mortality with TXA [40]. However, the latest study did not include the latest data and analyzed all patients enrolled in the CRASH-2 trial, including those with TBI and extracranial traumatic injuries [12]. Our conclusions are consistent with those of a recent study. Current perspectives suggest that the efficacy of TXA may depend on the severity of TBI, timing of TXA administration, and severity of extracranial hemorrhage, the advantages of which might be offset by the side effects of TXA [41]. In addition, patients with both impeding exsanguination and associated severe TBI are likely to be deceased prior to arrival at the emergency department, which can lead to selection bias in the process of data collection. Therefore, mortality can be affected by multiple factors.

In this study, we performed a subgroup analysis that included the timing of TXA administration, which did not change the results or conclusions for any of the outcomes of interest. Our results show that TXA had no effect on mortality. This is consistent with some research results [14, 15, 17, 18, 26]. However, this contrasts with the findings of a previous CRASH-3 trial, which claimed that TXA was safe in patients with TBI and that treatment within 3 h of injury reduced head injury-related death [42]. This conflicting result does not mean that administration within 3 h is ineffective for TBI patients because of the confounding effect of hemorrhage growth and TBI severity. Although the mortality of patients with TBI treated with TXA may be affected by multiple factors, the results of many large-scale RCTs, such as CRASH-3, indicated that absolute mortality reduction by TXA was low.

### Adverse event after the treatment of TXA with respect to time and dose

Thrombotic and neurological complications are the most common adverse events associated with TXA administration because of its antifibrinolytic activity and as a competitive antagonist of γ-aminobutyric acid (GABA) [9]. Coagulation disorders following TBI are associated with a complex interplay between coagulopathy, fibrinolysis, and hypercoagulability. A hypercoagulable state can promote the occurrence of different coagulation complications such as cerebral intravascular microthrombi or systemic disseminated intravascular coagulation. TXA, which blocks lysine-dependent plasmin generation and inhibits the dissolution and degradation of fibrin clots, can alter the delicate balance between coagulation and fibrinolysis and theoretically have detrimental implications for outcomes, resulting in vascular occlusive events, pulmonary embolism, and deep vein thrombosis [43]. TXA may not increase gastrointestinal bleeding during TBI [44]. Moderate to high doses (100 mg/kg/bolus and 10 mg/kg/h, for example) of TXA are potentially associated with neurological complications (seizures, transient ischemic attack, delirium) in adults and children [44–46]. TXA competitively inhibits glycine receptors in cortical and spinal cord neurons as well as GABA receptors in cortical and medullary neurons. Both inhibitory pathways of TXA cause an increased excitatory synaptic stimulus, which is theoretically prone to convulsion and stroke [11, 47].

In this meta-analysis, we analyzed various reported adverse events after treatment, including vascular occlusive events, deep vein thrombosis, neurological complications, gastrointestinal bleeding, myocardial infarction, infectious complications, and renal failure. No obvious adverse events related to TXA administration were found. The incidence of these events may be too low to demonstrate significant effects. Nevertheless, the possibility of bias should not be ruled out because the underlying pre-injury diseases of TBI patients were not fully recorded.

Our subgroup analysis showed that the timing of TXA administration was not a factor affecting adverse events. Notably, some researchers believe that the early use of TXA can effectively prevent adverse events [15, 21]. However, the most recent study did not include the latest data [12]. Our conclusions are consistent with those of a recent study. The study design was not a factor affecting the results of adverse events. This indicated that the experiment had low heterogeneity in different regions. The TXA dose was not a factor affecting adverse events. This indicated that different doses of TXA may have the same effect. However, there are few articles on TXA dose. Therefore, there is a risk of publication bias regarding TXA dose. In conclusion, using TXA to treat TBI patients should not be discontinued in clinical practice, solely due to the possibility of adverse events.

As has been mentioned above, this paper is the first study to investigate the efficacy of different time and dose of TXA in the treatment of TBI. There is no universal standard on the most effective dose and time of TXA administration, limiting its routine use in many centers. For TXA dose, the current conventional dose is 1 g TXA bolus followed by 1 g TXA maintenance, but a recent study showed 2 g TXA bolus followed by a placebo infusion [13]. Although different doses did not affect the results, the focus of this direction is a key direction of TXA treatment. For enrollment time of TXA, the traditional view is that enrollment time of TXA within 3 h has less complications [19, 25], but many patients still use TXA within 8 h due to long distance from the hospital or lack of drugs [20, 27]. Therefore, research on the efficacy of different time and dose of TXA in the treatment of TBI may greatly contribute to improving the TXA safety during the TBI treatment. In addition, the results of our meta-analysis showed that limiting the enrollment time of TXA within 3 h may be recommended. Specifically, the result of different time of TXA showed that using of TXA have no associated with all-cause mortality, all adverse events, the need of neurosurgical intervention and the number people of new bleeding. However, when enrollment time after trauma is less than 3 h, TXA can reduce mean blood volume (RR -0.50, 95% Cl [-0.94, -0.05], P 0.03). This shows that early enrollment time of TXA have a certain good effect, but the discover of specific benefits still need more clinical trials.

## Limitation

This study is the first to investigate the efficacy of different timings and doses of TXA for the treatment of TBI. We collected data from the latest studies and drew reliable conclusions. TXA at various times and doses was associated with reduced mean bleeding but not with mortality, adverse events, neurosurgical intervention, or rebleeding. However, our study had several limitations. First, the current study lacked the recorded time between injury and TXA delivery, which makes the timing of TXA inaccurate. The risk of publication bias cannot be excluded, even though Harbord’s test and Egger’s test showed *P* > 0.05. Only a few studies have reported the average time from injury to TXA administration and stratified them; hence, the results will be affected by covariates in the studies.

## Conclusion

TXA at different times and doses was associated with reduced mean bleeding but not with mortality, adverse events, neurosurgical intervention, or rebleeding. We need more research data on different detection indexes and levels of TXA in patients with TBI, as compared to those not receiving TXA; although the prognostic outcome for all harm outcomes was not affected, the potential for harm was not ruled out.

## Supplementary Information

**Additional file1:Appendix 1-1**. search strategy for medline , embase and pubmed. **Appendix 1-2**. Subgroup analysis (including study design (multisite RCT or single site RCT), Enrollment time after trauma (< 3h or > 3h), and TXA dose (2g TXA bolus followed by a placebo infusion or 1g TXA bolus followed by 1g TXA maintenance)).

## Data Availability

All data generated or analyzed during this study are included in this published article and its supplementary information files.

## Abbreviations

CI: Confidence interval
GABA: γ-Aminobutyric acid
GOS: Glasgow Outcome Scale
SMD: Standardized mean difference
TXA: Tranexamic acid
TBI: Traumatic brain injury
RCT: Randomized controlled trial
RR: Risk ratio
