# Supplementary material for: The efficacy of tranexamic acid treatment with different time and doses for traumatic brain injury: a systematic review and meta-analysis
Source: Thromb J. 2022 Dec 19;20:79. doi: 10.1186/s12959-022-00440-9 (PMC9762012; doi:10.1186/s12959-022-00440-9)
Supplement: Supplementary file 1 — Additional file1:Appendix 1-1. search strategy for medline , embase and pubmed. Appendix 1-2. Subgroup analysis (including study design (multisite RCT or single site RCT), Enrollment time after trauma (< 3h or > 3h), and TXA dose (2g TXA bolus followed by a placebo infusion or 1g TXA bolus followed by 1g TXA maintenance)). [file 12959_2022_440_MOESM1_ESM.docx]

**Appendix 1-1. Search strategy for MEDLINE , EMBASE AND PUBMED**

**Appendix 1-2. Subgroup analysis (including study design (multisite RCT or single site RCT), Enrollment time after trauma (< 3h or > 3h), and TXA dose (2g TXA bolus followed by a placebo infusion or 1g TXA bolus followed by 1g TXA maintenance))**

**Supplement Figure 10. Forest plot. Comparing TXA and placebo for the outcome of all-cause mortality in study design (multisite RCT or single site RCT)**

**Supplement Figure 11. Forest plot. Comparing TXA and placebo for all adverse events in study design (multisite RCT or single site RCT)**

**Supplement Figure 12. Forest plot. Comparing TXA and placebo for the need of neurosurgical intervention in study design (multisite RCT or single site RCT)**

**Supplement Figure 13. Forest plot. Comparing TXA and placebo for the number people of new bleeding in study design (multisite RCT or single site RCT)**

**Supplement Figure 14. Forest plot. Comparing TXA and placebo for mean hemorrhage volume in study design (multisite RCT or single site RCT)**

**Supplement Figure 15. Forest plot. Comparing TXA and placebo for the outcome of all-cause mortality in enrollment time after trauma (< 3h or > 3h)**

**Supplement Figure 16. Forest plot. Comparing TXA and placebo for all adverse events in enrollment time after trauma (< 3h or > 3h)**

**Supplement Figure 17. Forest plot. Comparing TXA and placebo for the need of neurosurgical intervention in enrollment time after trauma (< 3h or > 3h)**

**Supplement Figure 18. Forest plot. Comparing TXA and placebo for the number people of new bleeding in enrollment time after trauma (< 3h or > 3h)**

**Supplement Figure 19. Forest plot. Comparing TXA and placebo for mean hemorrhage volume in enrollment time after trauma (< 3h or > 3h)**

**Supplement Figure 20. Forest plot. Comparing TXA and placebo for the outcome of all-cause mortality in TXA dose (2g TXA bolus followed by a placebo infusion or 1g TXA bolus followed by 1g TXA maintenance)**

**Supplement Figure 21. Forest plot. Comparing TXA and placebo for all adverse events in TXA dose (2g TXA bolus followed by a placebo infusion or 1g TXA bolus followed by 1g TXA maintenance)**

**Supplement Figure 22. Forest plot. Comparing TXA and placebo for the need of neurosurgical intervention in TXA dose (2g TXA bolus followed by a placebo infusion or 1g TXA bolus followed by 1g TXA maintenance)**

**Appendix 1-1. Search strategy for MEDLINE , EMBASE AND PUBMED**

**Pubmed (inception to December 31^th^, 2021)**

--------------------------------------------------------------------------------

1 (("brain injuries, traumatic"[MeSH Terms] OR ("brain"[All Fields] AND "injuries"[All Fields] AND "traumatic"[All Fields]) OR "traumatic brain injuries"[All Fields] OR ("brain"[All Fields] AND "injuries"[All Fields] AND "traumatic"[All Fields]) OR "brain injuries, traumatic"[All Fields]) OR ("brain injuries, traumatic"[MeSH Terms] OR ("brain"[All Fields] AND "injuries"[All Fields] AND "traumatic"[All Fields]) OR "traumatic brain injuries"[All Fields] OR ("traumatic"[All Fields] AND "brain"[All Fields] AND "injury"[All Fields]) OR "traumatic brain injury"[All Fields])) OR ("brain injuries"[MeSH Terms] OR ("brain"[All Fields] AND "injuries"[All Fields]) OR "brain injuries"[All Fields] OR ("brain"[All Fields] AND "injury"[All Fields]) OR "brain injury"[All Fields])) OR TBI[All Fields]) OR ("head"[All Fields] AND "injury"[All Fields]) OR "head injury"[All Fields])) OR ("head"[All Fields] AND "trauma"[All Fields]) OR "head trauma"[All Fields])) (228,539)

2 "Tranexamic Acid"” or "TXA" or "AMCHA” or “AMCA” or “cyklokapron” or “kabi-2161” or “transamin” or “ugurol” or “t-amcha” (6,283)

3 ((randomized controlled trial[pt] OR controlled clinical trial[pt]) OR (randomized OR randomised OR randomly OR placebo[tiab]) OR (trial[ti]) OR (“Clinical Trials as Topic”[MeSH Major Topic])) NOT ((“Animals”[Mesh]) NOT (“Humans”[Mesh] AND “Animals”[Mesh])) (1,552,469)

4 1 and 2 and 3 (497)

**Ovid MEDLINE (inception to December 31^th^, 2021)**

--------------------------------------------------------------------------------

1 exp Brain Injuries/ (50123)

2 Craniocerebral Trauma/ (20765)

3 (traumatic brain adj (injur$ or concussion$ or contusion$ or laceration$)).tw. (18456)

4 (traumatic cerebral adj (injur$ or concussion$ or contusion$ or laceration$)).tw. (56)

5 (tbi or tbis).tw. (12567)

6 exp Intracranial Hemorrhages/ (58594)

7 ((brain or basal ganglia or cerebral or cranial or intracranial or posterior fossa or Subarachnoid) adj2 (bleed$ or h?emorrhag$)).tw. (30410)

8 Pituitary Apoplexy.tw. (779)

9 or/1-8 (147629)

10 Antifibrinolytic Agents/ (4010)

11 Tranexamic Acid/ (1982)

12 txa.tw. (933)

13 amca.tw. (329)

14 amcha.tw. (123)

15 amchafibrin.tw. (0)

16 amikapron.tw. (1)

17 amstat.tw. (0)

18 anexan.tw. (0)

19 antivoff.tw. (0)

20 caprilon.tw. (0)

21 (cl 65336 or cl65336).tw. (0)

22 cy?lo?apron.tw. (56)

23 exacyl.tw. (7)

24 fibrinon.tw. (0)

25 frenolyse.tw. (1)

26 hemostan.tw. (0)

27 hexa?apron.tw. (0)

28 kabi 2161.tw. (1)

29 kalnex.tw. (0)

30 lysteda.tw. (9)

31 micranex.tw. (0)

32 rikaparin.tw. (0)

33 ronex.tw. (0)

34 spotof.tw. (0)

35 t-amcha.tw. (51)

36 theranex.tw. (0)

37 tramic.tw. (0)

38 tranex.tw. (0)

39 tranexam.tw. (9)

40 tranexa?ic acid.tw. (1907)

41 tranexic.tw. (0)

42 trans achma.tw. (0)

43 trans amcha.tw. (19)

44 transamin.tw. (18)

45 transexamic acid.tw. (5)

46 traxamic.tw. (0)

47 trenaxin.tw. (0)

48 ugurol.tw. (5)

49 or/10-48 (7906)

50 9 and 49 (388)

**Embase (inception to December 31^th^, 2021)**

--------------------------------------------------------------------------------

1 exp brain injury/ (112797)

2 head injury/ (36287)

3 (traumatic brain adj (injur$ or concussion$ or contusion$ or laceration$)).tw. (24818)

4 (traumatic cerebral adj (injur$ or concussion$ or contusion$ or laceration$)).tw. (52)

5 (tbi or tbis).tw. (19172)

6 exp brain hemorrhage/ (82666)

7 ((brain or basal ganglia or cerebral or cranial or intracranial or posterior fossa or Subarachnoid) adj2 (bleed$ or h?emorrhag$)).tw. (39870)

8 Pituitary Apoplexy.tw. (902)

9 or/1-8 (229865)

10 antifibrinolytic agent/ (3841)

11 tranexamic acid/ (6543)

12 txa.tw. (527)

13 amca.tw. (271)

14 amcha.tw. (90)

15 amchafibrin.tw. (19)

16 amikapron.tw. (3)

17 amstat.tw. (1)

18 anexan.tw. (0)

19 antivoff.tw. (0)

20 caprilon.tw. (9)

21 (cl 65336 or cl65336).tw. (1)

22 cy?lo?apron.tw. (435)

23 exacyl.tw. (103)

24 fibrinon.tw. (0)

25 frenolyse.tw. (13)

26 hemostan.tw. (0)

27 hexa?apron.tw. (11)

28 kabi 2161.tw. (4)

29 kalnex.tw. (0)

30 lysteda.tw. (33)

31 micranex.tw. (0)

32 rikaparin.tw. (0)

33 ronex.tw. (0)

34 spotof.tw. (6)

35 t-amcha.tw. (33)

36 theranex.tw. (0)

37 tramic.tw. (0)

38 tranex.tw. (17)

39 tranexam.tw. (12)

40 tranexa?ic acid.tw. (2691)

41 tranexic.tw. (0)

42 trans achma.tw. (0)

43 trans amcha.tw. (5)

44 transamin.tw. (59)

45 transexamic acid.tw. (12)

46 traxamic.tw. (0)

47 trenaxin.tw. (0)

48 ugurol.tw. (44)

49 or/10-48 (9852)

50 9 and 49 (698)

**COCHRANE Controlled Clinical Trials Registry**

--------------------------------------------------------------------------------

#1 MeSH descriptor: [Tranexamic Acid] explode all trees (1236)

#2 MeSH descriptor: [Craniocerebral Trauma] explode all trees (3938)

#3 MeSH descriptor: [Brain Injuries] explode all trees (2612)

#4 MeSH descriptor: [Intracranial Hemorrhages] explode all trees (2098)

#5 ((brain or basal ganglia or head or "cerebro-cranial" or cerebrocranial or "intra-cranial" or crani* or cerebral or intracranial or posterior fossa or subarachnoid) adj2 (trauma$ or concussion$ or wound$ or contusion$ or laceration$ or injur* or h?emorrhage* or h?ematoma$)).ti,ab,kw. (4603)

#6 (Trauma* adj encephal*).ti,ab,kw. (31)

#7 (tbi or tbis or pituitary apoplexy).ti,ab,kw. (69)

#8 #2 or #3 or #4 or #5 or #6 or #7 or #8 (9880)

#9 #1 and #8 (79)

**Google Scholar**

tranexamic AND (brain or basal ganglia or head or cranium or cerebral or intracranial or posterior fossa or subarachnoid) (120)

# **Table 1 Baseline characteristics of included studies**

| study autor and years | study design | patients TXA/placebo | TXA dose | male(%) | Mean or median age in years TXA/placebo | Enrollment time after trauma | inclusion criterial | exclusion criterial |
| --- | --- | --- | --- | --- | --- | --- | --- | --- |
| Roberts et al.2019 | Multisite RCT | 6406/6331 | 1g TXA bolus followed by 1g TXA maintenance | TXA: 3742 (80%) placebo: 3660 (80%) | TXA:41.7(19) placebo:41.9(19) | 2h | (1) Adults with TBI within 3 h of injury (2) GCS ≤ 12 or any intracranial bleeding on CT scan | Major extracranial bleed |
| Rowell et al.2020 | Multisite RCT | 657/309 | Bolus-Maintenance arm: 1g TXA bolus followed by 1g TXA. Bolus only arm: 2g TXA bolus followed by a placebo infusion | Bolus-Maintenance: 227(73%) Bolus-Only: 255(74%) placebo: 233(75%) | Bolus-Maintenance arm: 39(26-57) Bolus Only arm: 40(26-56) Placebo arm: 36(25-55) | 2h | (1) GCS ≤ 12 (2) Prehospital SBP ≥ 90 (3) Age ≥ 15yrs (or weight ≥ 50 kg if age is unknown) | (1) GCS = 3 with unreactive pupil (2) CPR by EMS prior to randomization (3) Burns (4) Pregnancy |
| Mahmood et al.2021 | Multisite RCT | 884/883 | 1g TXA bolus followed by 1g TXA maintenance | TXA: 701(79%) placebo: 712(81%) | TXA:45(29-64) placebo: 45(29-63) | 3h | (1) Adults with head injury who were within 3 hours of injury; (2) Glasgow Coma Score (GCS) of ≤12 ; (3) any intracranial bleeding on CT, and no significant extracranial bleeding | significant extracranial bleeding |
| van Wessem et al.2021 | Single site RCT | 120/114 | 1g TXA bolus followed by 1g TXA maintenance | TXA: 80(67%) placebo: 77(68%) | TXA:42 (23–59) placebo: 53 (33–65) | 1h | TBI (AIS head ≥ 3) who were admitted to the adult ICU | AIS head scores based on isolated C-spine injuries |
| Mojallal et al.2020 | Single site RCT | 56/44 | 1g TXA bolus followed by 1g TXA maintenance | TXA: 40(71.4%) placebo: 40(90.9%) | N/A | 8h | (1)age>18. (2)detection of cerebral hemorrhage in brain CT scan (3)passage of less than 8 hours after trauma incidence (4)negative history of taking anticoagulants (5)negative history of blood coagulation system impairments | patients who underwent craniotomy less than 24 hours |
| Mousavinejad et al.2020 | Single site RCT | 20/20 | 1g TXA bolus followed by 1g TXA maintenance | TXA: 6(30%) placebo: 8(40%) | 55 ± 19/55 ± 18 | 8h | (1) ≥ 18 years within 8 h of injury (2) TBI on brain CT with no significant epidural hemorrhage (3) The need for surgery | (1) Pregnancy (2) Coagulopathy (3) Massive transfusion and/or fresh frozen plasma (FFP) |
| Yutthakasemsunt et al.2013 | Single site RCT | 120/118 | 1g TXA bolus followed by 1g TXA maintenance | TXA: 103(86%) placebo: 107(91%) | 35 (16)/ 34 (15) | 8h | (1) Age ≥ 16 years (2) Moderate to severe TBI (GCS) 4 to 12 (3) Had a CT brain within 8 h (4) No immediate indication for surgery | (1) Immediate need for surgery (2) Coagulopathy (3) Known to be receiving a medication that affects hemostasis (4) Pregnancy |
| Fakharian et al.2017 | Single site RCT | 78/78 | 1g TXA bolus followed by 1g TXA maintenance | TXA: 67(90.5) placebo: 66(88) | 42 ± 18/39 ± 18 | 8h | (1) Age ≥ 15 years (2) Non-penetrating injury and any kind of Traumatic ICH (3) Arrived at the hospital within 8 h 4) No need for brain surgery during the first 8 h | (1) Major organ damage (2) Pregnancy (3) Receiving any medication that disturbs homeostasis (4) Coagulopathy |
| Jokar et al.2017 | Single site RCT | 40/40 | 1g TXA bolus followed by 1g TXA maintenance | TXA: 32(40.0%) placebo: 28(35.0%) | 35 ± 15/ 36 ± 145 | 2h | (1) TBI patients aged 15 years and more (2) Within 2 h of injury onset (3) Acute ICH (volume of less than 30 ml) based on CT scan findings | (1) GCS < 8 (2) Need for surgery (3) Cerebral edema with midline shift (4) Coagulation disorders (5) Pregnancy (6) History or current VTE |
| Ebrahimi et al.2019 | Single site RCT | 40/40 | 1g TXA bolus followed by 1g TXA maintenance | SDH-TXA: 17(85%) SDH-placebo: 17(85%) EDH-TXA: 16(80%) EDH-placebo: 18(90%) | SDH: 40 ± 18/40 ± 18 EDH: 24 ± 7/25 ± 7 | 8h | (1) Adults within 8 h of injury (2) Isolated SDH or EDH requiring surgery | (1). Major extracranial bleeding (2) Massive transfusion (3) Coagulopathy (4) Pregnancy |
| Perel et al.2012 | Multisite RCT | 133/137 | 1 g TXA bolus followed by 1 g TXA maintenance | TXA: 111(84.0) placebo: 117(85.0) | 36.2 (14.0)/37.0 (13.7) | 8h | 1) Fulfils the inclusion criteria for the CRASH-2 trial (2) GCS ≤ 14 (3) Baseline clinical CT scan consistent with TBI | (1) Pregnancy and (2) Patients for whom a second brain CT scan was not possible |
| [Bossers et al.2021](https://pubmed.ncbi.nlm.nih.gov/?term=Bossers+SM&cauthor_id=33284310) | Multisite RCT | 693/1134 | 1g TXA bolus followed by 1g TXA maintenance: 615  >2g TXA bolus: 4 | TXA: 486 (70%) placebo: 797 (70%) | 47 (25-66)/45 (22-65) | N/A | 1)severe TBI, GCS≤8 2)suspected rather than confirmed TBI because prehospital treatment, including administration of tranexamic acid | 1)BRAIN-PROTECT database who were not transported to a participating trauma center (no follow-up data were available) 2)undergoing prehospital traumatic cardiopulmonary resuscitation (inherently very high mortality, regardless of treatment) |
| chakroun et al.2018 | Single site RCT | 96/84 | 1g TXA bolus followed by 1g TXA maintenance | TXA: 88 (91.7%) placebo: 797 (70%) | 44 ± 20/39 ± 18 | 8h | (1)age>18. (2)intracranial bleeding in the first or the second brain CT-scan (3)a delay of management in the study centre under 24 h after trauma | 1)significant extra cranial bleeding 2)TXA can improve outcome |

1. *RCT* randomized-controlled trial, *TXA* tranexamic acid, *GCS* Glasgow Coma Scale, GOS Glasgow Outcome Scale , *TBI* traumatic brain injury, *SDH* subdural hemorrhage, *EDH* epidural hemorrhage,  *ICH* intracranial hemorrhage, *N/A* not applicable

**Appendix 1-2. Subgroup analysis (including study design (multisite RCT or single site RCT), Enrollment time after trauma (< 3h or > 3h), and TXA dose (2g TXA bolus followed by a placebo infusion or 1g TXA bolus followed by 1g TXA maintenance))**

**Study design (multisite RCT or single site RCT)**

**Supplement Figure 10. Forest plot. Comparing TXA and placebo for the outcome of all-cause mortality in study design (multisite RCT or single site RCT)**

**
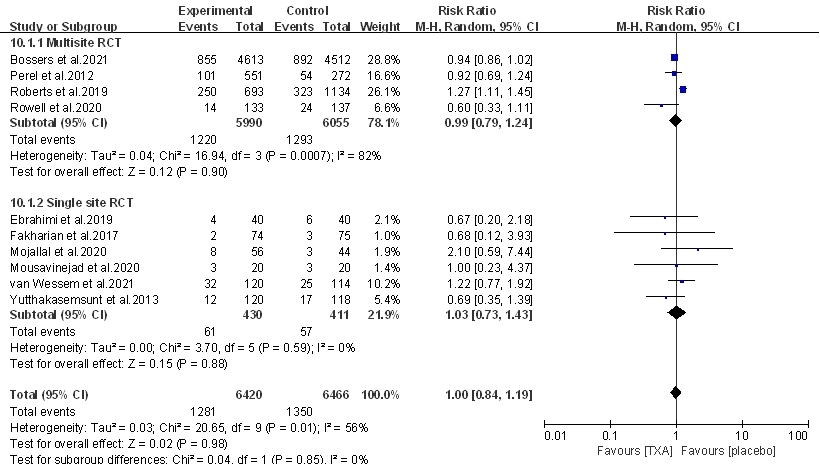
**

**Supplement Figure 11. Forest plot. Comparing TXA and placebo for all adverse events in study design (multisite RCT or single site RCT)**

**
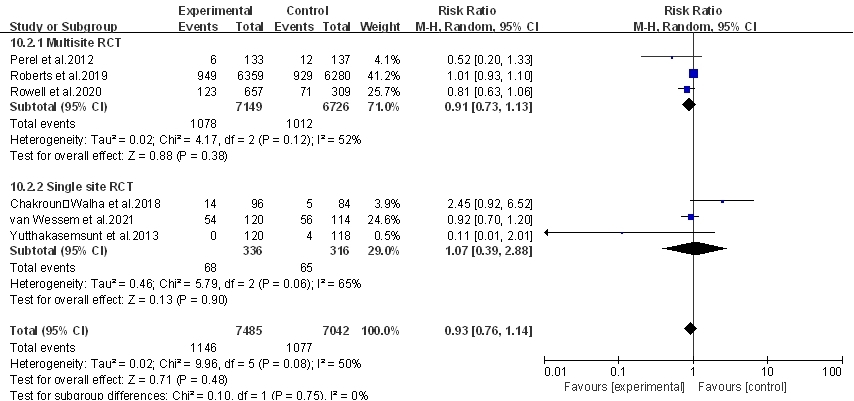
**

**Supplement Figure 12. Forest plot. Comparing TXA and placebo for the need of neurosurgical intervention in study design (multisite RCT or single site RCT)**

**
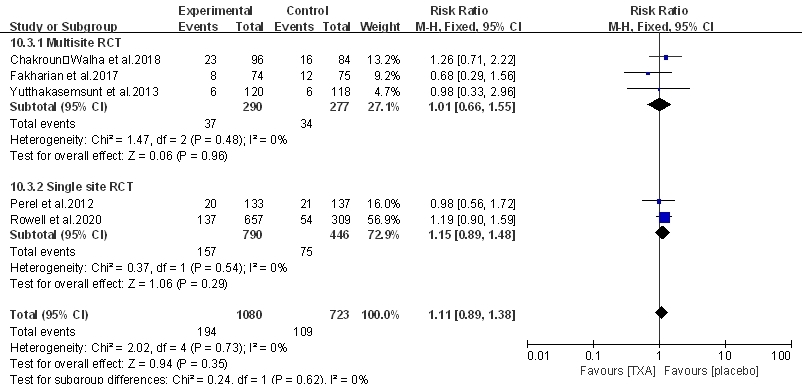
**

**Supplement Figure 13. Forest plot. Comparing TXA and placebo for the number people of new bleeding in study design (multisite RCT or single site RCT)**

**
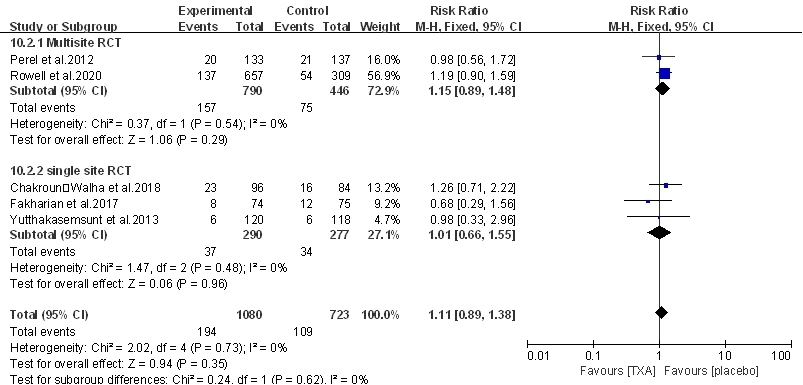
**

**Supplement Figure 14. Forest plot. Comparing TXA and placebo for mean hemorrhage volume in study design (multisite RCT or single site RCT)**

**
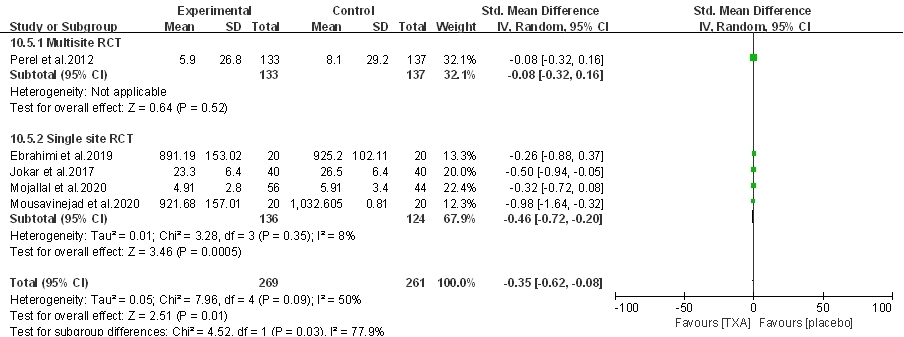
**

**Supplement Figure 15. Forest plot. Comparing TXA and placebo for the outcome of all-cause mortality in enrollment time after trauma (< 3h or > 3h)**

**
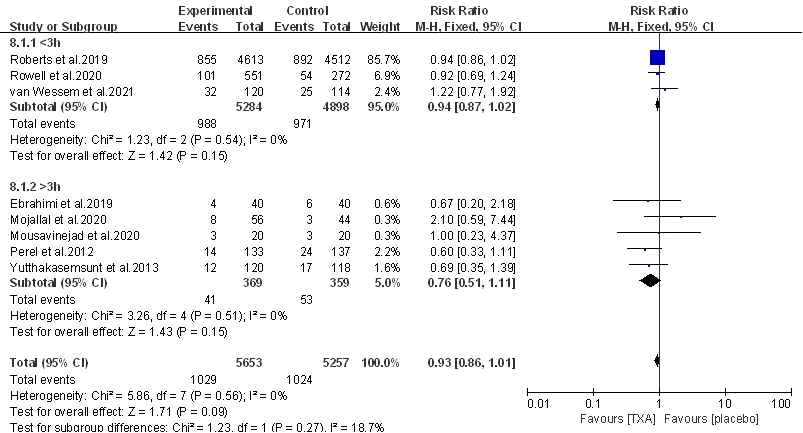
**

**Supplement Figure 16. Forest plot. Comparing TXA and placebo for all adverse events in enrollment time after trauma (< 3h or > 3h)**

**
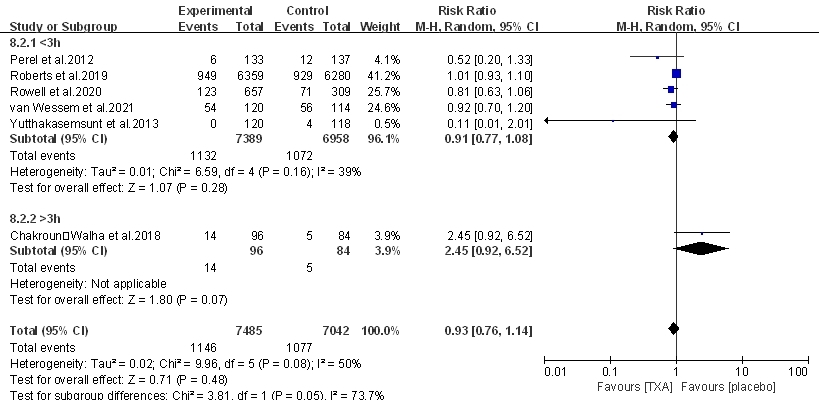
**

**Supplement Figure 17. Forest plot. Comparing TXA and placebo for the need of neurosurgical intervention in enrollment time after trauma (< 3h or > 3h)**

**
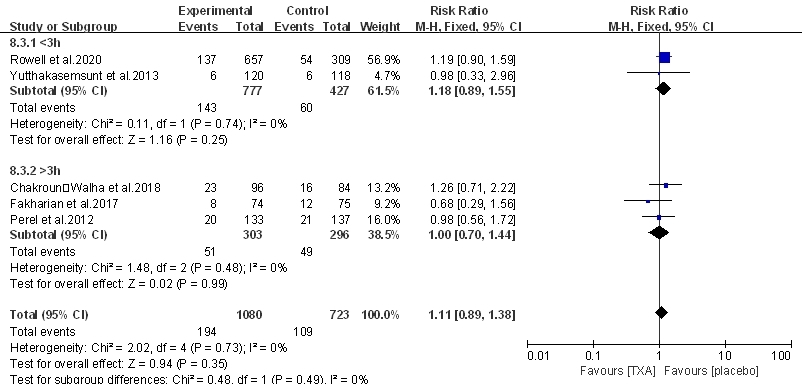
**

**Supplement Figure 18. Forest plot. Comparing TXA and placebo for the number people of new bleeding in enrollment time after trauma (< 3h or > 3h)**

**
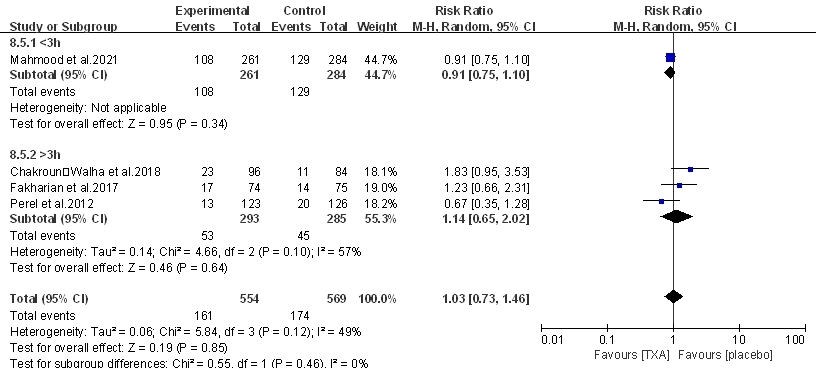
**

**Supplement Figure 19. Forest plot. Comparing TXA and placebo for mean hemorrhage volume in enrollment time after trauma (< 3h or > 3h)**

**
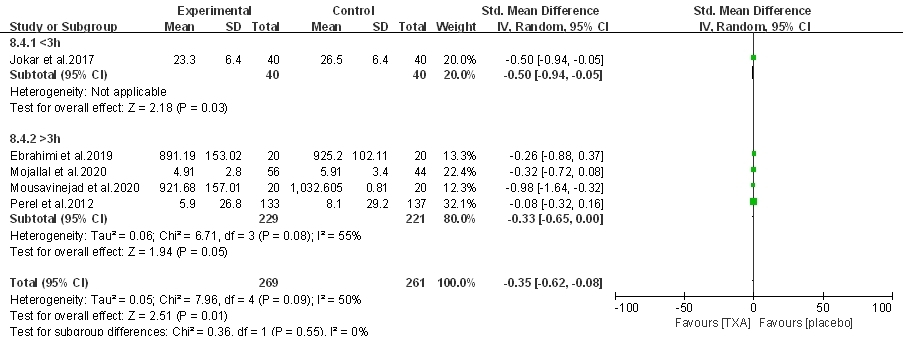
**

**Supplement Figure 20. Forest plot. Comparing TXA and placebo for the outcome of all-cause mortality in TXA dose (2g TXA bolus followed by a placebo infusion or 1g TXA bolus followed by 1g TXA maintenance)**

**
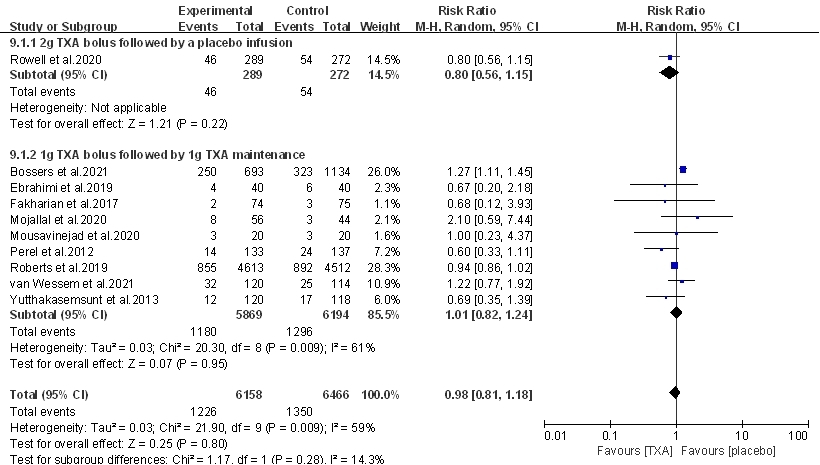
**

**Supplement Figure 21. Forest plot. Comparing TXA and placebo for all adverse events in TXA dose (2g TXA bolus followed by a placebo infusion or 1g TXA bolus followed by 1g TXA maintenance)**

**
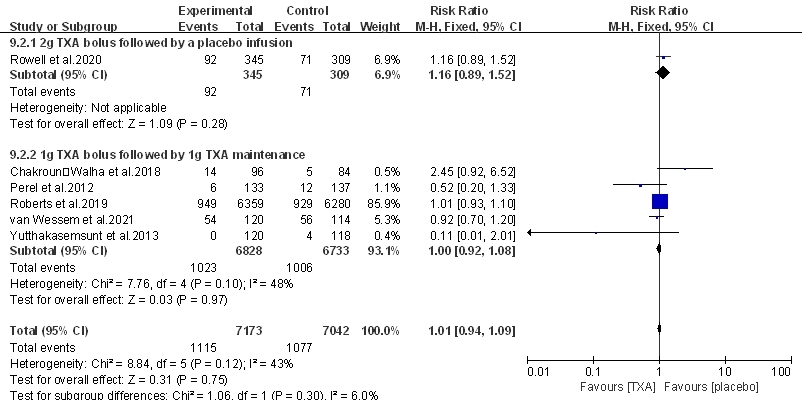
**

**Supplement Figure 22. Forest plot. Comparing TXA and placebo for the need of neurosurgical intervention in TXA dose (2g TXA bolus followed by a placebo infusion or 1g TXA bolus followed by 1g TXA maintenance)**

**
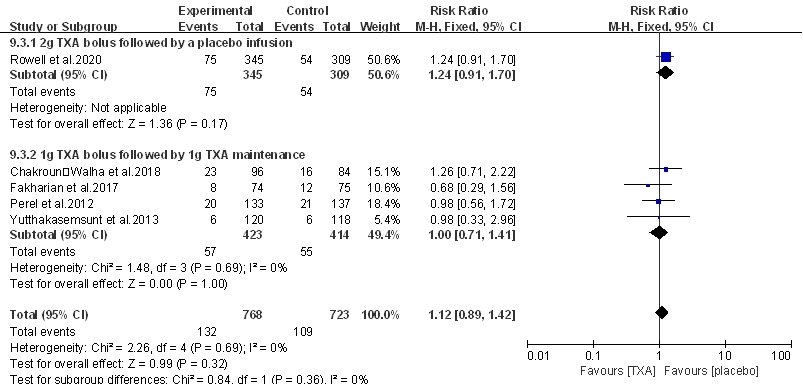
**
